# Self-report and parent-report of physical and psychosocial well-being in Dutch adolescents with type 1 diabetes in relation to glycemic control

**DOI:** 10.1186/1477-7525-5-10

**Published:** 2007-02-16

**Authors:** Maartje de Wit, Henriette A Delemarre-van de Waal, Jan Alle Bokma, Krijn Haasnoot, Mieke C Houdijk, Reinoud J Gemke, Frank J Snoek

**Affiliations:** 1 Department of Medical Psychology, VU University Medical Center Amsterdam, The Netherlands; 2 EMGO Institute, VU University Medical Center Amsterdam, The Netherlands; 3 Department of Pediatrics, VU University Medical Center Amsterdam, The Netherlands; 4 Department of Pediatrics, Spaarne Ziekenhuis Hoofddorp, The Netherlands; 5 Department of Pediatrics, Medical Center Alkmaar, The Netherlands; 6 Department of Pediatrics, Juliana Kinderziekenhuis Den Haag, The Netherlands

## Abstract

**Background:**

To determine physical and psychosocial well-being of adolescents with type 1 diabetes by self-report and parent report and to explore associations with glycemic control and other clinical and socio-demographic characteristics.

**Methods:**

Demographic, medical and psychosocial data were gathered from 4 participating outpatient pediatric diabetes clinics in the Netherlands. Ninety-one patients completed the Child Health Questionnaire-CF87 (CHQ-CF87), Centre for Epidemiological Studies scale for Depression (CES-D), and the DFCS (Diabetes-specific Family Conflict Scale). Parents completed the CHQ-PF50, CES-D and the DFCS.

**Results:**

Mean age was 14.9 years (± 1.1), mean HbA_1c _8.8% (± 1.7; 6.2–15.0%). Compared to healthy controls, patients scored lower on CHQ subscales role functioning-physical and general health. Parents reported less favorable scores on the behavior subscale than adolescents. Fewer diabetes-specific family conflicts were associated with better psychosocial well-being and less depressive symptoms. Living in a one-parent family, being member of an ethnic minority and reporting lower well-being were all associated with higher HbA_1c _values.

**Conclusion:**

Overall, adolescents with type 1 diabetes report optimal well-being and parent report is in accordance with these findings. Poor glycemic control is common, with single-parent families and ethnic minorities particularly at risk. High HbA_1c _values are related to lower social and family functioning.

## Background

Adolescence is a period of rapid biological changes accompanied by increasing physical, cognitive and emotional maturity that can seriously complicate diabetes regulation. Indeed, adolescents with type 1 diabetes as a group display the worst glycemic control compared to other age-groups [1,2], which puts them at increased risk for developing complications [3,4]. From a developmental perspective, the burden diabetes places on routines and friendships can compromise emotional and social well-being, adversely affecting quality of life (QoL). Finding the right balance between good psychosocial functioning and preserving long-term health by maintaining near normal blood glucose values is a challenge for adolescents with diabetes and their families, as well as their care providers.

Different results are found when comparing diabetic adolescents with their healthy peers. Diabetic adolescents tend to report no differences or even better QoL compared to healthy peers [5-9]. Only one study found adolescents with diabetes reporting worse psychosocial health [10]. Parents of diabetic adolescents, however, do tend to rate their adolescents' health worse as compared to parents of healthy adolescents [5-7,9,10]. Direct comparisons of adolescent and parent reported QoL scores are rarely made in diabetes research.

The association of glycemic control with QoL by either adolescent or parent report is inconsistent across studies. Half of the studies investigating the relationship found an association between lower well-being and higher HbA_1c _values [6-14], while the other half did [7,12,15-19] not. There are a few longitudinal studies into the psychosocial well-being in adolescents with diabetes, that indicate that behavioral problems and physical functioning are important in influencing later glycemic control [6,11]. More family conflicts appear to associate with lower QoL scores in adolescents with diabetes [7,17].

Here we present data from a cross-sectional study on physical and psychosocial well-being of Dutch adolescents with type 1 diabetes and the relationship with clinical parameters, which were gathered at baseline as part of an ongoing randomized controlled trial (RCT) testing the effectiveness of periodic monitoring of Health-related Quality of Life (HRQoL) during outpatient visits. These data allow us to 1) compare reported health status of adolescents with diabetes with healthy peers, 2) examine concordance between adolescent and parent report and 3) explore associations with socio-demographic characteristics and glycemic control. A better understanding of these issues is pivotal to improve quality of care for teenagers with diabetes and optimize clinical outcomes.

## Methods

Participants in the age range 13–17 were recruited from four pediatric diabetes outpatient clinics in the Netherlands, including one academic center (VUmc), by sending an information letter. The study was approved by the Medical Ethical committees of all participating centers and written informed consent was obtained from patients and parents. Those children who did not return the informed consent form were approached by their pediatrician at the next routine appointment and were given the opportunity to participate in the study at that time if they so wished. No time or not interested were the most mentioned reasons for declining participation. All adolescents and their parents received a booklet with questions regarding demographic information and questionnaires to assess physical and psychosocial well-being which they could complete at home and return by mail or in the waiting area at their routine appointment. Height, weight, most recent HbA_1c _and treatment regimen were recorded form the charts.

## Measures

**Physical and Psychosocial well-being **of the adolescents was measured using the 87-item child report version of the Child Health Questionnaire (CHQ-CF87), covering domains of physical, emotional, social and mental health [20]. This questionnaire consists of 10 multi-item subscales, 4 single item scales and two summary subscales, psychosocial and physical health. Ratings of all scales are based on children's functioning over the previous 4 weeks. Parents completed the 50-items parent form of the CHQ-PF50, analogue to the CHQ-CF87. It consists of 11 multi-item and 4 single item subscales and two summary subscales, psychosocial and physical health. All scale scores are transformed to a range of 0 – 100, with higher scores indicating better well-being.

**Depression **As part of psychosocial well-being we assessed the depressive symptomology of both adolescents and parents with the 20-item Centre for Epidemiological Studies scale for Depression (CES-D) [21], scored from 0 to 3 on the basis of frequency of depressive symptoms reported in the past two weeks, from never to daily. Total CES-D summation scores range between 0 (no depressive symptoms) to 60 (most frequent/severe depressive symptoms).

The CES-D was initially developed to measure symptoms of depression in adult community studies but has been used in adolescent (diabetes) populations subsequently [22-24]. Similar to the US National Longitudinal Study of Adolescent Health and the SEARCH for diabetes in youth study we stratified depression severity as "minimal" (0–15), "mild" (16–23), and "moderate/severe" (≥24) [23-25]. For parent report we used the conventional cut-off score of ≥ 16 to define likely cases of depression.

**Diabetes-specific family conflict **Each adolescent and parent completed the adapted version by Laffel et al. of the Diabetes-specific Family Conflict Scale to assess the degree of family conflict on 19 management tasks [26,27]. In this measure, the level of family conflict was rated on a 3-point scale (1 = never argue, 2 = argue a fair amount and 3 = always argue). Previous reports showed excellent reliability in both child and parent responses [7,26]. The scores could range from 19 to 57, with 57 indicating conflict on all items.

### Statistical analyses

Substitution of missing values and calculation of the subscale scores was performed according to the manual of the CHQ-CD87 and CHQ-PF50 [20]. We examined differences in the CHQ-CF87 between boys and girls, between healthy adolescents and adolescents with diabetes and between adolescents with diabetes and their parents score on the CHQ-PF50 using analysis of variance (ANOVA) or, in case of non-normal distribution, Kruskall-Wallis or Mann-Whitney U-tests. Spearman correlations were used to examine agreement between adolescents and parents. Differences in characteristics between adolescents with optimal (HbA_1c _< = 7.5%) and sub-optimal glycemic control (HbA_1c _> 7.5%) were examined using χ^2^- and Mann-Whitney U-tests. Correlations between HbA_1c_, the CHQ subscales, CES-D and DFCS were explored with Pearson and Spearman correlation coefficients. Multi-linear regression was used to correlate HbA_1c _with physical and psychosocial well-being. To correct for demographic and diabetes-related variables, we first entered age, sex, ethnicity, family structure, diabetes duration and hospital, after that forward regression was used for the physical and psychosocial well-being variables and interaction terms. SPSS version 11.0.1 was used to execute all analyses.

## Results

Of the total 171 eligible subjects, 91 adolescents with type 1 diabetes and their parents consented to participate in the RCT. No differences were found in age, gender or HbA_1c _as between participating and non-participating adolescents, the latter however where more likely to be of another ethnicity and from 1 particular hospital.

As shown in Table 1, mean age was 14.9 ± 1.1 years and mean diabetes duration 6.6 ± 4.1 years. BMI was higher in girls (21.8 kg/m^2^) than in boys (20.4 kg/m^2^) (P = 0.04). More boys (29.3 %) than girls used three insulin injections per day (P = 0.04), while more girls (17.1 %) then boys used a pump (P = 0.04). Of all the adolescents participating in the study, 18.7 % lived in a single parent family. Ten participants were of another ethnicity (11 %), half of them of Moroccan descent. The distribution of education levels was comparable to the general Dutch school population.

**Table 1:** Demographic and diabetes related information

	Participants*	Non-participants*
Number patients	91	80
Sex (boys/girls)	47/44	43/37
Age (range)	14.9 ± 1.1 (13 – 16.5)	15.0 ± 1.1 (13 – 16.5)
BMI	21.1 ± 3.2	-
Diabetes duration	6.4 ± 4.2	-
HbA_1c _(%) (range)	8.8 ± 1.7 (6.2 – 15.0)	9.0 ± 1.5 (5.5 – 13.8)
Injections per day (%)		-
2	8.0	
3	42.0	
4	35.2	
pump	14.8	
Single parent families (%)	18.7	-
living with father (mother)	2.2 (16.5)	
Caucasian (%)	89	-

Data are means ± SD, unless otherwise indicated. * No significant differences between participants and non-participants regarding sex, age or HbA_1c_; no information is available for the other variables.

### Physical and psychosocial well-being

Physical and psychosocial well-being reported by either adolescents or parents did not differ on adolescent's age, ethnicity, diabetes duration, BMI or treatment regimen. Adolescents living in a one-parent family reported more limitations in activities with friends and school work due to behavioral problems (role functioning-behavioral subscale CHQ-CF87) (P = 0.035). Boys only rated their physical functioning (P = 0.02) and bodily pain (P = 0.03) better and their global behavior (P = 0.01) worse as compared to girls. No further differences were found between boys and girls.

**Figure 1:**
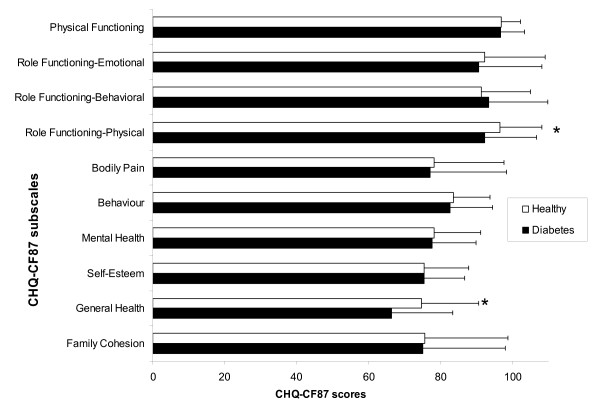
**Mean CHQ-CF87 scores of adolescents with diabetes and healthy adolescents**. Mean CHQ-CF87 scores of adolescents with diabetes as compared to healthy adolescents with standard deviation. Higher scores indicate better well-being. * Significant difference between adolescents with diabetes and healthy adolescents: P < 0.01. Not all subscales are available for healthy adolescents.

When comparing the scores of the adolescents in our study to scores from a Dutch school population [28], we found that adolescents with diabetes reported lower scores on the role functioning-physical subscale (mean difference = -4.3, P = 0.006). This suggests that diabetic adolescents experience more limitations in activities with friends and school work due to physical problems. Also, our patients reported their general health to be worse (mean difference = -8.3, P < 0.001) (Figure 1).

Interestingly, the scores of the adolescent – parent pairs are largely concordant, as illustrated by the correlations for directly comparable subscales ranging from r = 0.27 for the mental health subscale (P = 0.004) to r = 0.70 for the family activities subscale (P < 0.001). Adolescents rated less behavioral problems (higher scores) than their parents did (P = 0.001), which translated in a significant difference in the psychosocial health summary scale (P = 0.004).

As expected, depression scores (CES-D) correlated negatively with the scores for all CHQ-CF87 subscales, ranging from r = -0.20 for change in health to r = -0.67 for the psychosocial summary score (Table 2). Six adolescents (3 boys) (6.6%) had scores indicative of mild depression (16 and 23), while three adolescents (all boys) (3.3%) scored 24 or above, indicating moderate/severe depression. There were no differences in CES-D scores between boys and girls.

**Table 2:** Correlations between CHQ scores and HbA_1c_, Depression (CES-D) and Diabetes Family Conflict (DFCS) scores

**CHQ-CF87/PF50**		**HbA**_1c_	**CES-D child**	**DFCS child**	**DFCS parent**
**Physical Health (summary score)**	child	-0.20	-0.53**	-0.20	
	parent	-0.30**	-0.33**		-0.20
Physical Functioning	child	0.05	-0.24*	0.012	
	parent	-0.08	-0.40**		0.01
Role functioning- Physical	child	-0.05	-0.37**	-0.18	
	parent	-0.17	-0.29**		0.02
Bodily Pain	child	-0.003	-0.24*	-0.07	
	parent	-0.10	-0.27**		0.06
General Health	child	-0.26*	-0.44**	-0.28**	
	parent	-0.31**	-0.16		-0.40**
Change in health	child	-0.08	-0.20	-0.12	
	parent	-0.01	-0.27**		-0.19
**Psychosocial Health (summary score)**	child	-0.40**	-0.67**	-0.39**	
	parent	-0.34**	-0.36**		-0.32**
Role functioning					
Emotional#	child	-0.17	-0.37**	-0.32**	
Behavioral#	child	-0.33**	-0.40**	-0.31**	
Emotional/Behavioral†	parent	0.085	-0.33**		-0.19
Behavior	child	-0.26*	-0.59**	-0.39**	
	parent	-0.23*	-0.24*		-0.29**
Mental Health	child	-0.21*	-0.61**	-0.26*	
	parent	-0.21*	-0.25*		-0.32**
Self Esteem	child	-0.14	-0.38**	-0.19	
	parent	-0.18	-0.32**		-0.15
Parent Impact Emotion†	parent	-0.17	-0.34**		-0.36**
Parent Impact Time†	parent	-0.36**	-0.30**		-0.17
Family Activities	child	-0.34**	-0.66**	-0.51**	
	parent	-0.34**	-0.38**		-0.28**
Family Cohesion	child	-0.16	-0.36**	-0.26*	
	parent	-0.08	-0.25*		-0.15
**Diabetes Family Conflict Scale**	child	0.20	0.40**	-	0.50**
	parent	0.20	0.13	0.50**	-
**CES-D (depression)**	child	0.35**	-	0.40**	0.13

#Adolescent form only, †Parent form only, * Significant at P < 0.05, ** significant at P < 0.01.

Of the parents, thirteen (14.4%) reported scores indicating likely depression (≥16). Adolescents with parents scoring above 16 on the CES-D (indication for depression), did not report more depressive symptoms as compared to adolescents with non-depressive parents. Those 13 parents scoring above the cut-off score of 16, rated the physical (P = 0.005) and psychosocial (P = 0.043) health of their children to be worse compared to parents with no depressive symptoms (CHQ-PF50 subscales: role functioning-emotion/behavioral P = 0.025, role functioning-physical P = 0.006, bodily pain P = 0.030, family activities P = 0.034).

### Diabetes-specific family conflict

As could be expected, more diabetes-specific family conflicts were associated with lower psychosocial well-being and more depressive symptoms (Table 2). Parents and adolescents largely agreed on the topics of conflict (r = 0.50, P < 0.001). 'Logging blood sugar results', 'remembering to check blood sugars' and 'meals and snacks' were the most mentioned issues.

### Glycemic control

Mean HbA_1c _was 8.8% (± 1.7; 6.2–15.0%), with 81% of the adolescents above the recommended 7.5% [29]. HbA_1c _was not significantly correlated with age, gender, diabetes duration, treatment regimen or BMI. Adolescents with good glycemic control (≤7.5%) reported less family conflicts (P = 0.046) than the others. All adolescents from a one-parent family (18.7%) were among the poorly controlled (> 7.5%), as were all adolescents of another ethnicity (11 %). As shown in Table 2, higher HbA_1c _values were associated with more depressive symptoms and lower psychosocial well-being.

A linear regression was conducted to explore predictors of glycemic control, showing a significant association of HbA_1c _with single parent family (B = 1.31, P = 0.001), other ethnicity (B = 1.43, P = 0.002) and the CHQ-CF87 subscale role functioning-behavioral (B = -.04, P = 0.001) (R^2 ^= .45, P < 0.001). In other words: living in a one-parent family or being of another ethnicity is associated with a raise in HbA_1c _of respectively 1.31 % and 1.43 %. Reporting 10 points less limitations on school work and activities with friends due to behavioral difficulties is associated with a decrease in HbA_1c _of 0.4%.

## Discussion

Findings from our study show that adolescents in general function well, i.e. comparable to healthy peers, in concert with previous reports [5-9]. We found moderate to high rates of adolescent – parent agreement, especially for the physical well-being subscales. It has been suggested that parent-child agreement is higher for chronically ill children, compared to parents and their healthy children, and that agreement between parents and their adolescents is higher for physical functioning [30]. Adolescents only rated less behavioral problems than their parents did. This difference was suggested in two other studies in adolescents with diabetes as well; however those studies also found differences in other subscales [6,31]. As there are no Dutch norm scores available for parents of healthy adolescents, the difference in behavior may be related to diabetes or may be an effect of puberty per se. Our findings are somewhat different from previous studies in adolescents with diabetes, which found lower agreement rates and parents reporting their child's well-being to be worse than parents of healthy children [5-7,9-11]. The high agreement in our study could be due to the overall high levels of well-being of the adolescents, with little room for disagreement due to the ceiling effect.

In line with the relatively high CHQ scores, we found the prevalence of depression in our sample not to be elevated. This contrasts with studies reporting two- to threefold higher rates of depression in teenagers with diabetes [12,24,32]. This maybe related to our age range, where most of our patients are under 16 years old and therefore at lower risk of depression than older adolescents [33].

In both parent and adolescent report, lower psychosocial well-being is associated with more depressive symptoms and diabetes-specific family conflicts. This is in accordance with earlier studies which suggest that diabetes-specific family factors are strongly related to quality of life in youth with diabetes [7,17]. Depression is found to be associated with more family conflicts, as well as with lower well-being [12,18,34,35]

In more than 80 % of the adolescents in our study, diabetes is suboptimal controlled, defined by HbA_1c _levels above 7.5 %. Mean HbA_1c _levels are in accordance with previous studies in adolescents, the distribution, however, is not further specified in most publications. If a looser criterion of 8.0 % is used, still 66 % is poorly controlled. Persisting high levels of HbA_1c _throughout puberty will significantly increase their risk of developing complications [3]. Our data suggest that adolescents of another ethnicity and those living in single parent families are especially at risk for deterioration of glycemic control, as has been shown in other studies [13,36]. Moreover, we found more depressive symptoms and family conflicts as well as lower psychosocial well-being to be associated with higher HbA_1c _levels. The strongest association when taking into account demographic and clinical variables was found for those adolescents reporting more limitations in activities with friends and schoolwork due to behavioral difficulties.

Studies investigating the relationship between physical and psychosocial well-being and glycemic control have reported mixed results [6-19], with few longitudinal studies suggesting lower physical and behavioral functioning to be predictive of poor glycemic control [6,11]. Studies linking depressive symptoms and HbA_1c _also report inconsistent findings [32], although more recent literature [12,24,34,35] does suggest that more depressive symptoms are associated with higher HbA_1c _values, as in our study. More and larger longitudinal studies are needed to better understand the complex relationship between psychological functioning and HbA_1c _in adolescents with type 1 diabetes. Clearly, our study points to the need to address psychosocial issues as integral part of outpatient diabetes care, particularly for poorly controlled adolescents. Psychological, family and educational interventions have shown to be effective in improving well-being and glycemic control in diabetic adolescents and their families, although results are not consistent across all studies [37,38]. The future will tell whether monitoring HRQoL as part of periodic outpatient visits, as we are currently testing in a RCT, will help to improve clinical outcomes.

A limitation of our study is the fact that about half of the patients decided not to participate in the RCT, possibly causing positive selection bias. However, poor glycemic control was obvious not a reason for decline. Besides that, the variation among individual adolescents in physical and psychosocial well-being is quite large suggesting that adolescent with good as well as with low well-being participated in our study.

## Conclusion

In conclusion, Dutch adolescents with type 1 diabetes receiving secondary care overall report a satisfactory quality of life, while diabetes control is suboptimal for the majority of them. The participants seem to have found a balance between an acceptable level of daily diabetes self-management and QoL. The challenge then for health care professionals is to help these young patients and their families to further improve glycemic control without diminishing subjective well-being. Psychosocial risk factors for poor glycemic control were identified, underscoring the importance of a holistic approach to diabetes, particularly in this vulnerable age-group.

## Abbreviations

CES-D Centre for Epidemiological Studies scale for Depression

CHQ-CF87 Child Health Questionnaire-Child Form 87 item

CHQ-PF50 Child Health Questionnaire-Parent From 50 items

DFCS Diabetes Family Conflict Scale

HRQoL Health-related Quality of Life

QoL Quality of Life

## Competing interests

The author(s) declare that they have no competing interests.

## Authors' contributions

MW has coordinated the research, collected and analysed the data and drafted the manuscript, FS, RG and HD have participated in the design of the study, interpreting the results and helped to draft the manuscript, JAB, KH and MH have contributed to the acquisition of the data and the draft of the manuscript. All authors read and approved the final manuscript.
